# Carbon ion radiotherapy combined with immunotherapy: synergistic anti-tumor efficacy and preliminary investigation of ferroptosis

**DOI:** 10.1007/s00262-023-03544-x

**Published:** 2023-09-30

**Authors:** Qingting Huang, Jiyi Hu, Li Chen, Wanzun Lin, Jing Yang, Weixu Hu, Jing Gao, Haojiong Zhang, Jiade Jay Lu, Lin Kong

**Affiliations:** 1 Department of Radiation Oncology, Shanghai Proton and Heavy Ion Center, Shanghai, 201321 China; 2 Department of Radiation Oncology, Shanghai Proton and Heavy Ion Center, Fudan University Cancer Hospital, Shanghai, 201321 China; 3 Shanghai Key Laboratory of Radiation Oncology (20dz2261000), Shanghai, 201321 China; 4 Shanghai Engineering Research Center of Proton and Heavy Ion Radiation Therapy, Shanghai, 201321 China; 5 Department of Radiation Oncology, Proton and Heavy Ion Center, Heyou International Hospital, Foshan, 528000 China

**Keywords:** Carbon ion radiotherapy, Immunotherapy, Anti-tumor efficacy, Ferroptosis

## Abstract

Carbon ion radiotherapy (CIRT) may yield satisfactory clinical outcomes for patients who are resistant to radiotherapy. However, the therapeutic impact of carbon ions is still limited in certain recurring or refractory tumors. Therefore, we aimed to evaluate the synergistic anti-tumor effects of immune checkpoint inhibitors (ICIs) in combination with CIRT. We then explored the involvement of ferroptosis in a preliminary investigation. A tumor-bearing mouse model was established, and mice were inoculated subcutaneously with B16-OVA cells into the flanks of both hind legs. Mice were assigned to four groups to receive CIRT, ICIs, or combined treatment. Thereafter, we conducted transcriptome sequencing (RNA-seq), bioinformatics analysis, and various immune-related experiments on the available tumor tissues to investigate differences in the synergistic anticancer effects and potential mechanisms across the groups. The combination therapies significantly improved the survival of mice and inhibited tumor growth, both at local and distant sites. Based on bioinformatics and RNA-seq data, immune-related pathways and genes, immune cell infiltration, and the production of cytokines and chemokines were the most enhanced in the combined treatment group compared to other groups. Finally, we identified a potential role for ferroptosis in the development of local anti-tumor synergy during CIRT combination treatment. In conclusion, this study showed that CIRT and ICIs can enhance the anti-tumor immune effects. We also proposed that ferroptosis may induce anti-tumor effects in CIRT combination therapy, offering a unique perspective on its ability to enhance immunotherapy responses.

**Supplementary Information:**

The online version contains supplementary material available at 10.1007/s00262-023-03544-x.

## Introduction

Radiotherapy (RT) is one of the main cancer treatment modalities and may be used alone or in conjunction with other therapies to increase response rates and promote better survival rates. [1] Radiotherapy can act as a radiogenic in situ vaccine, causing immunogenic cell death (ICD) and immunogenic modulation, which activates the immune cells that mediate radiation-caused tumor-specific cell death and tumor cell DNA damage. [2, 3] The RT immunomodulatory potential, especially when applied in combination with immunological checkpoint inhibitors (ICIs), enhances the immunosuppressive status while synergistically improving the immune response, thus providing new perspectives for systemic treatment strategies against refractory cancer. [4, 5] Additionally, preclinical and clinical research revealed the synergistic effects of radiation combined with anti-PD-1/PD-L1 antibodies and/or anti-CTLA-4 antibodies in tumor treatment. [6–8] These results suggest that combination treatments may be more effective in treating some resistant cancers.

The most cutting-edge and efficient RT technique available today is particle beam radiotherapy, specifically for carbon ion RT (CIRT), which offers physical and biological advantages compared to conventional photon radiation for cancer treatment. [9] CIRT causes systemic anti-tumor immunity, primes the tumor microenvironment, and causes stronger tumor immunogenicity than photon therapy, which may contribute to increasing tumor responsiveness to ICI therapy. [10, 11] In recent animal studies, the combination of CIRT and ICI treatment was more efficient in eliminating both locally irradiated tumors and distantly unirradiated cancers, demonstrating that the use of carbon ions in combination with immunotherapy may have a synergistic anticancer impact. [12, 13] However, data validating the use of carbon ions in combination with immunotherapy are lacking, and the mechanism underlying these treatments is unclear. Excessive lipid peroxidation induces ferroptosis, an iron-dependent cell death type. [14] Ferroptosis is a key anti-tumor mechanism in photon radiation, immunotherapy, and even photon radiotherapy and immunotherapy combination. [15–18] However, the ferroptosis function in carbon ion radiation alone or conjunction with immunotherapy for synergistic anti-tumor activity is unclear.

Herein, we first assessed the synergistic anti-tumor effectiveness of carbon ion irradiation in collaboration with immunotherapy for both local and distant cancers in vivo and then demonstrated synergistic immunity activation using combination treatment. Thereafter, we conducted a preliminary investigation into ferroptosis participation in carbon ion radiation combined with ICIs. This study will advance the clinical use of carbon ion radiation in combination with immunotherapy.

## Materials and methods

The details of the method are also presented in the supplementary material.

### Cell line experiments

The mouse B16-OVA cell line used here was purchased from the American Type Culture Collection (ATCC). The murine melanoma cell line B16 gave rise to the OVA-transfected clone known as B16-OVA.

### Mouse tumor model experiments

The SPHIC's ethical committee authorized all animal experimentation protocols and techniques. We obtained female, six- to eight-week-old C57BL/6 mice from the Shanghai SLAC Laboratory Animal Company. For experiments involving tumor-bearing mice, animals were subcutaneously injected with 5 × 10^5^ B16-OVA cells into the flanks of both hind legs to initiate tumor formation. On days 0, 7, and 14 after radiation treatment, mice in the ICI and combined groups received intraperitoneal injections of 200 µg of anti-PD-L1 and anti-CTLA-4 antibodies. A rat immunoglobulin G (IgG) isotype antibody was applied as a control. After implanting subcutaneous tumor cells, mice in the anti-CD8 antibody group received 150 µg of anti-CD8 antibody intraperitoneally every four days. After radiation therapy, mice in the ferroptosis inhibitor liproxstatin-1 groups received daily intraperitoneal injections of 30 mg/kg liproxstatin-1.

### Treatment planning and delivery of CIRT

Single-fraction CIRT (4 Gy, physical dosage) was administered to tumors on the right hind legs of the mice (right tumor: irradiated tumor; left tumor: unirradiated tumor). Radiation was delivered according to previously described methods. [19] The energy of the carbon beams ranged from 118.41 to 140.01 MeV/u, and the mean dose averaged linear energy transfer (LET) within the SOBP was 96.94 keV/um.

### RNA sequencing analysis

RNA differential expression analysis was performed using DESeq2 [20] R package between two different groups. The genes/transcripts with the parameter of false discovery rate (FDR) below 0.05 and absolute fold change ≥ 2 were considered differentially expressed genes (DEGs)/transcripts.

### Bioinformatics analysis of RNA sequencing data

GSVA R package was used to quantify the signaling pathways in each group, as identified by their enrichment in the MSigDB collection (c2.kegg.v7.1 symbols.gmt; h.all.v7.0.cymbols.gmt). The enrichment score was presented using a heatmap.

The relationship between DEG expression and immune cell infiltration between different treatment groups was analyzed using the single sample gene set enrichment analysis (ssGSEA) method from R package GSVA (version 3.6). These GSEA enrichment scores were then used for each immune cell type that was obtained from each sample and was completed using “GSVA” and “GSEA’’ as the immune cell infiltration measure in each sample. ESTIMATE was used to calculate the immune score, which is the estimate of immune cells in tumor tissue calculated using the “estimate” R package (version 3.6).

### Multi-cytokine assay

Briefly, tumor tissue was homogenized and cell supernatants from each therapy group were collected for a multi-cytokine test. Cytokine levels were evaluated using a Luminex 200 system (Luminex) using a panel of 31 mouse cytokines (LX-MultiDTM-31) following the manufacturer’s recommendations. Tumors were collected for multi-cytokine tests 8 days after radiation treatment.

### Statistical analysis

Two-tailed unpaired Student's *t *tests or Wilcoxon tests were used to compare the two groups. Two-way ANOVA was used when comparing more than two groups. To determine the *p* values for Kaplan–Meier survival curves, the log-rank test was used. Data are provided as the mean ± SEM. R was used for all bioinformatics statistical calculations (v.3.6.3). The asterisks depicted in the figures indicate significance as follows: *p < 0.05, **p < 0.01, ***p < 0.001, ****p < 0.0001.

## Results

### Local anti-tumor effects following CIRT combined with ICIs

**Fig. 1:**
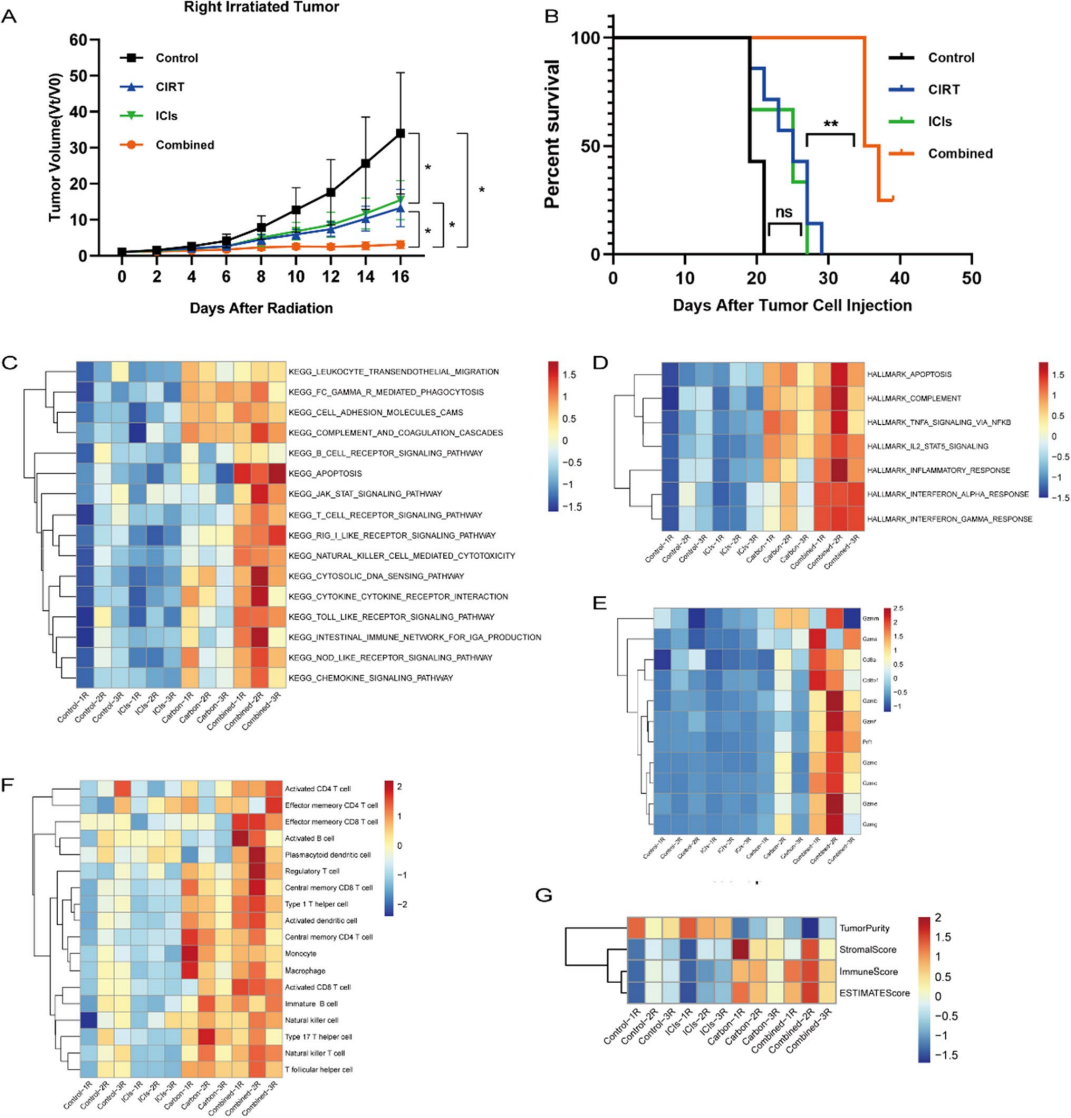
Local anti-tumor effects following carbon ion radiotherapy (CIRT) combined with immune checkpoint inhibitors (ICIs), and the results of bioinformatics analyses based on transcriptome sequencing (RNA-seq) data of irradiated tumors. **A** The progression and fold changes of the local irradiated tumor sizes and a combination of treatments dramatically inhibited tumor development compared to other groups. **B** Mice in the combined group displayed the longest survival times. **C**, **D** KEGG and HALLMARK analysis showed that immune-related pathways were noticeably enriched in the combined treatment group. **E** Enrichment of immune-related genes in each group, which may indicate the function of tumor-infiltrating CD8 + T cells. **F**, **G** Immune cell infiltration in each group observed using bioinformatics analysis, indicating that a combination of CIRT with ICIs may be able to improve anti-tumor immunity and immune cell infiltration. ssGSEA is used to validate the variation in immune cell infiltration in each group and identified higher immune cell infiltration levels in the combined treatment group than in the other groups. The stromal score, immune score, and estimate score for tumor samples in each group were determined using the ESTIMATE algorithm, with the combination treatment group displaying the highest ESTIMATE scores. Tumor collection for RNA-seq was performed 8 days after radiation treatment

We originally developed a mouse tumor model on both hind legs to test whether a CIRT and ICI combination exhibits more favorable outcomes and synergistic benefits as an anticancer treatment. After receiving treatments, the irradiated tumor development delay was assessed in mice. Figure 1A, B depicts the local irradiated tumor progression, fold changes in tumor sizes, and mice survival during the observation period. CIRT alone, ICIs, and combination treatment reduced mouse tumor growth rates compared to the control group. Importantly, the combination of treatments dramatically increased tumor development inhibition. Mortality was considered when the tumor volume exceeded 1500 mm^3^. The mice in the combined group displayed the longest survival times, demonstrating the potential synergy of anti-tumor responses induced by carbon ion radiation and immunotherapy.

### Bioinformatics analysis based on transcriptome sequencing (RNA-seq) data

We conducted transcriptome sequencing (RNA-seq) on the available irradiation tumor tissues to investigate differences in the synergistic anticancer effects across the groups. Thereafter, bioinformatics analysis was performed to determine enriched immune-related or differential pathways. Our analysis revealed that immune-related pathways were noticeably enriched in the combined treatment group compared to other groups (Fig. 1C, D), suggesting that CIRT significantly influences anti-tumor immunity with potential synergy when used in combination with ICIs. Immune-related genes, such as *CD8A*, *CD8B*, *GZMA*, *GZMB*, and *PRF1,* that may indicate the function of tumor-infiltrating CD8 + T cells were considerably enriched in the group receiving combination therapy (Fig. 1E). Moreover, we used ssGSEA and ESTIMATE algorithm to validate the variation in immune cell infiltration in each group (Fig. 1F and G) and identified higher immune cell infiltration levels in the combined treatment group than in the other groups. Therefore, a combination of CIRT with immunotherapy might improve anti-tumor immunity.

### Lymphocyte infiltration of irradiated tumors

**Fig. 2:**
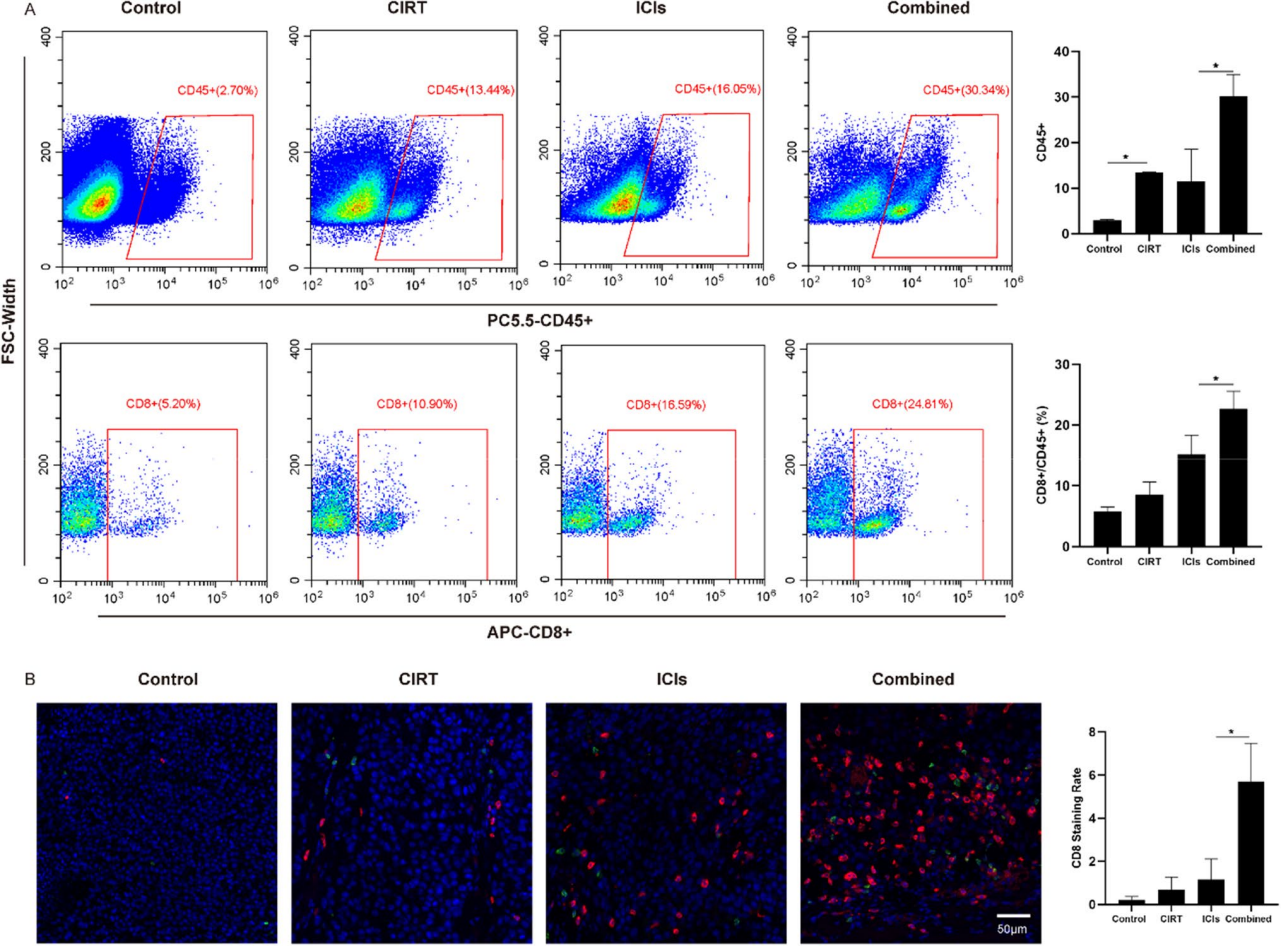
The CD8 + cell infiltration of irradiated tumors in each group. **A** Flow cytometry analysis demonstrated that tumors of mice in the combination therapy group displayed the most prominent CD45 + and CD8 + cell infiltration. **B** Analysis using immunofluorescence assay (blue: DAPI; CD4: green; CD8: red). Immunofluorescence assays also showed that CD8 + infiltrates remained abundant in the tumors following combination therapy. Tumor collection and detection for CD8 + cell infiltration was performed 8 days after radiation treatment

Tumor-infiltrating immune cells play a crucial role in the anti-tumor immune response. Flow cytometry was used to measure immune cell infiltration into tumors after CIRT, ICI, and combination treatment to corroborate the results from bioinformatics analyses regarding the immune cell infiltration level. Tumors from combination therapy mice displayed the most prominent CD45 + and CD8 + cell infiltration (Fig. 2A). The tumor growth delay of each group suggests that CIRT and ICIs would exert a synergistic immunological impact to boost immune cell infiltration in tumor tissues and trigger tumor retreat. Immunofluorescence assays were performed to identify CD4 + (green) and CD8 + (red) T cell infiltration, which revealed that CD8 + infiltrates remained abundant in tumors following combination therapy (Fig. 2B). These findings agree with the results obtained from bioinformatics analysis based on the data from transcriptome sequencing.

### Cytokine and chemokine secretion in irradiated tumors

**Fig. 3:**
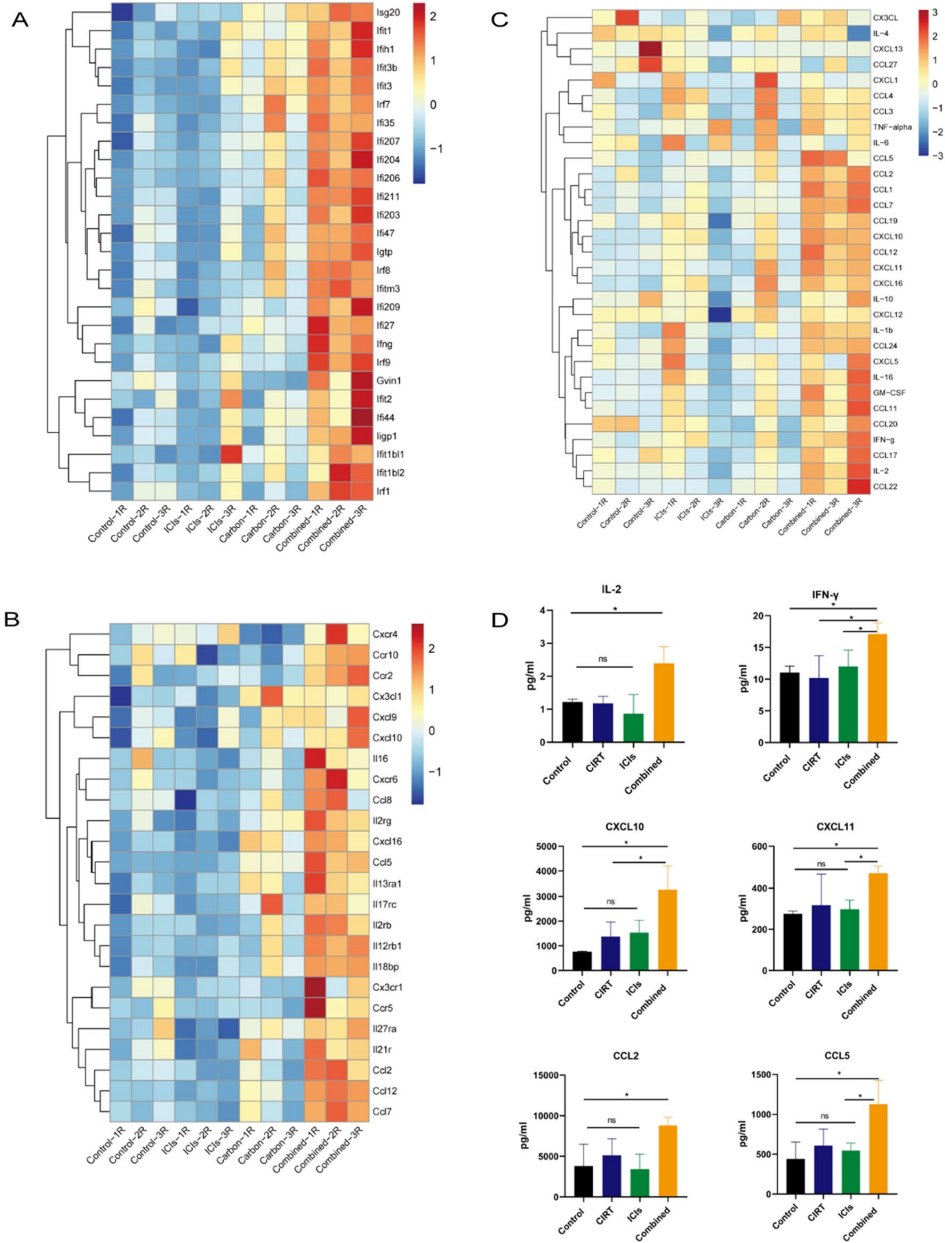
Cytokine and chemokine secretion in irradiated tumors. **A**, **B** The enrichment of interferon response and cytokine and chemokine genes in each group using bioinformatics analysis. **C**, **D** Cytokine and chemokine secretion of irradiated tumors using the multi-cytokine test. Tumor collection and detection for cytokine and chemokine secretion was performed 8 days after radiation treatment

The immune microenvironment may be altered through cytokine and chemokine administration. Transcriptome sequencing data revealed that the combined therapy group had elevated inflammatory and interferon responses and cytokine and chemokine gene enrichment (Figs. 1D and 3A, B). Using a multi-cytokine test, we further detected the secretion of numerous cytokines and chemokines in irradiated tumor tissues compared to the control group following CIRT, ICI, and combination therapy. Cytokines and chemokines were considerably enhanced in the radiation tumor microenvironment following combination treatment (Fig. 3C, D).

### Abscopal effects following CIRT combination with ICIs

**Fig. 4:**
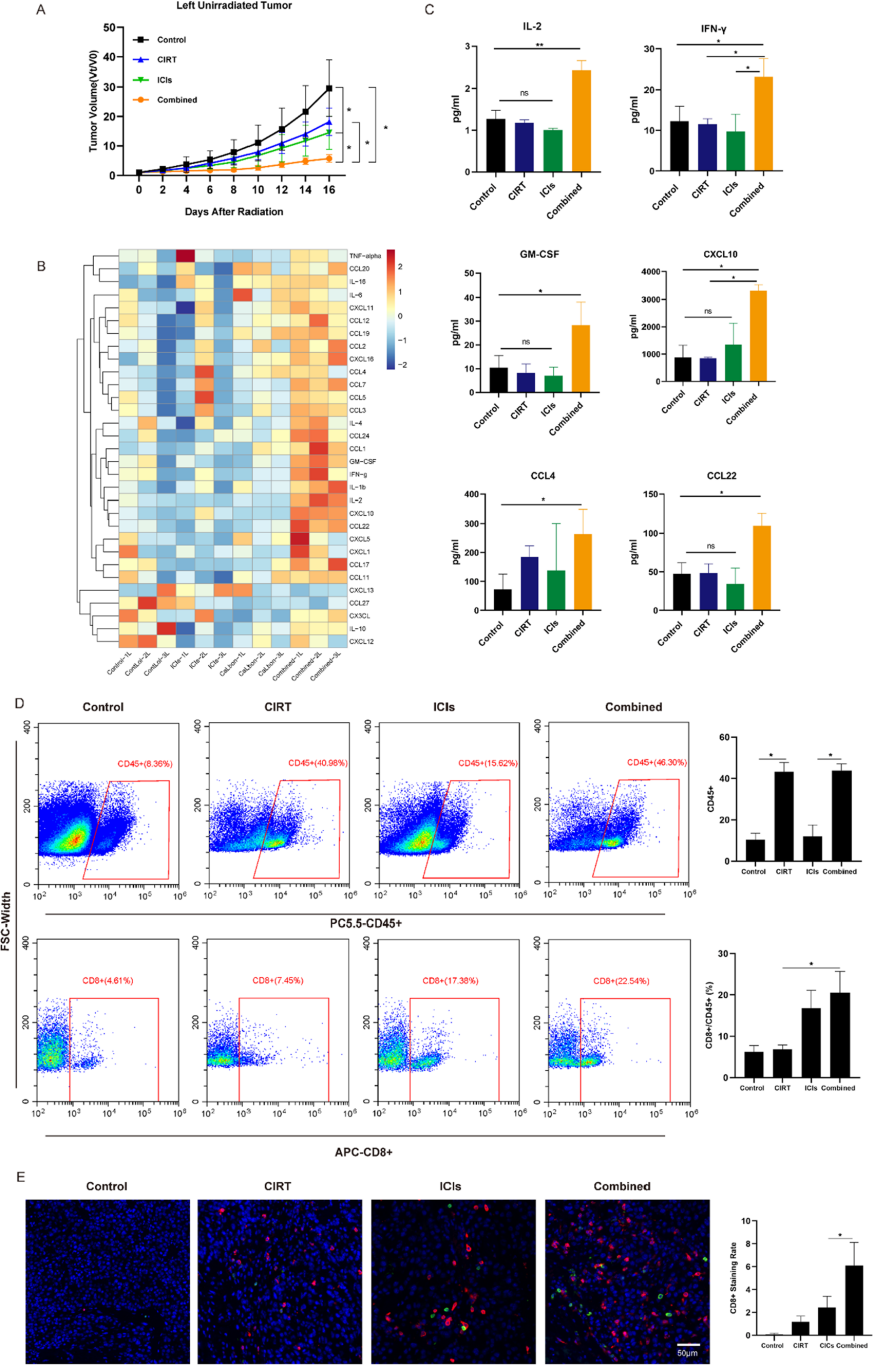
Abscopal effects following CIRT combined with ICIs. **A** The progression and fold changes of the unirradiated tumor sizes. **B**, **C** Cytokine and chemokine secretion in unirradiated tumors detected using the multi-cytokine test. **D** Flow cytometry results demonstrated that tumors of mice in the combination therapy group displayed the most prominent CD45 + and CD8 + cell infiltration. **E** Immunofluorescence assay results (blue: DAPI; CD4: green; CD8: red) showed that the CD8 + infiltrates remained abundant in the unirradiated tumors following combination therapy. Tumor collection and detection for CD8 + cell infiltration was performed 8 days after radiation treatment

Radiation and immunotherapy combination may enhance the abscopal impact. Mice exposed to localized CIRT with ICIs displayed an abscopal effect. Figure 4A shows unirradiated tumor development and fold changes in mouse tumor volumes during the observation period. The tumors of the unirradiated mice in the control group grew faster than those of mice in other groups, whereas tumor development was greatly suppressed in the combination therapy group. Similar to local tumor growth inhibition, which was most pronounced in the combination group, distant tumor growth was also inhibited. Next, the multi-cytokine test was used to identify cytokines and chemokine production and found combination treatment was associated with the highest levels of these immune response-related mediators (Fig. 4B, C). Finally, flow cytometry and immunofluorescence were conducted to confirm that the combined therapy groups had a higher immune cell infiltration rate in the unirradiated tumors (Fig. 4D, E). These results strengthen our finding that the immunological effects induce the abscopal effect.

### CD8 + T cell depletion reverses the anti-tumor effect

**Fig. 5:**
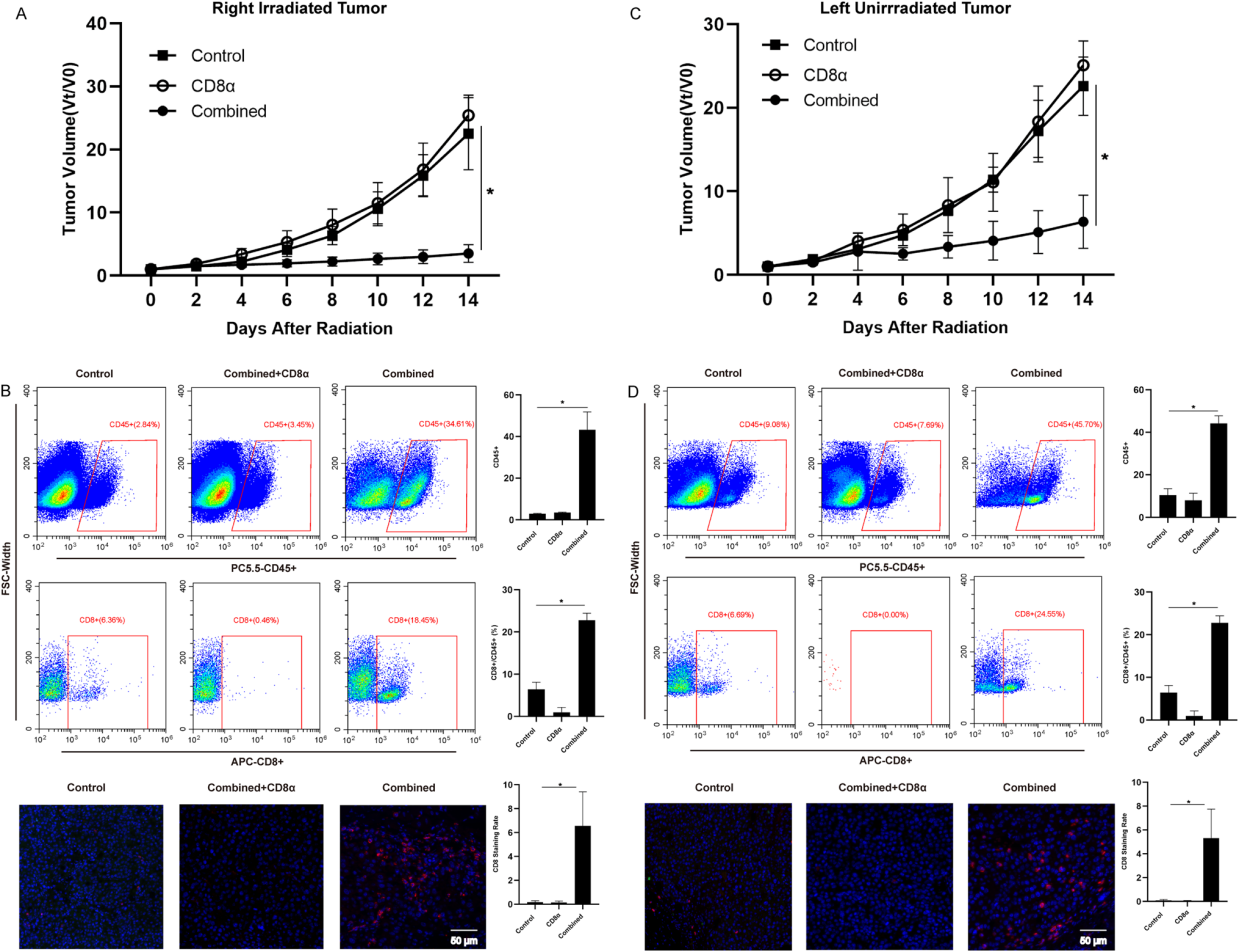
CD8 + T cell depletion reverses the anti-tumor effect. **A** The progression and fold changes of irradiated and unirradiated tumor sizes after CD8 + T cell depletion. **B** CD8 + T cell infiltration of irradiated tumors after CD8 + T cell depletion. **C** CD8 + T cell infiltration of unirradiated tumors after CD8 + T cell depletion

Given the significance of CD8 + T cells in mediating tumor cell eradication, the CD8 cell function on the immune response was evaluated using a mouse tumor model and an anti-CD8 monoclonal antibody as part of the combination treatment. Figure 5A and C indicates the growth of the irradiated and unirradiated tumors and the fold changes in the tumor volumes in the control, combination, and combination plus anti-CD8 monoclonal antibody groups during the observation period. The tumor-suppressive impact of carbon ions coupled with immunotherapy in both irradiated and unirradiated tumors was obviously hindered by CD8 cell elimination using anti-CD8 antibodies. The degree of CD8 + cell infiltration was verified using flow cytometry and immunofluorescence to ascertain whether anti-CD8 monoclonal antibodies reduce the number of CD8 + cells. Both irradiated tumors (Fig. 5B) and unirradiated tumors showed significant CD8 + cell depletion (Fig. 5D). These results demonstrate the crucial roles that CD8 + cytotoxic T cells play in the combination therapy-induced anti-tumor response (local and abscopal effects).

### Ferroptosis is involved in the synergistic efficacy of combination therapy

**Fig. 6:**
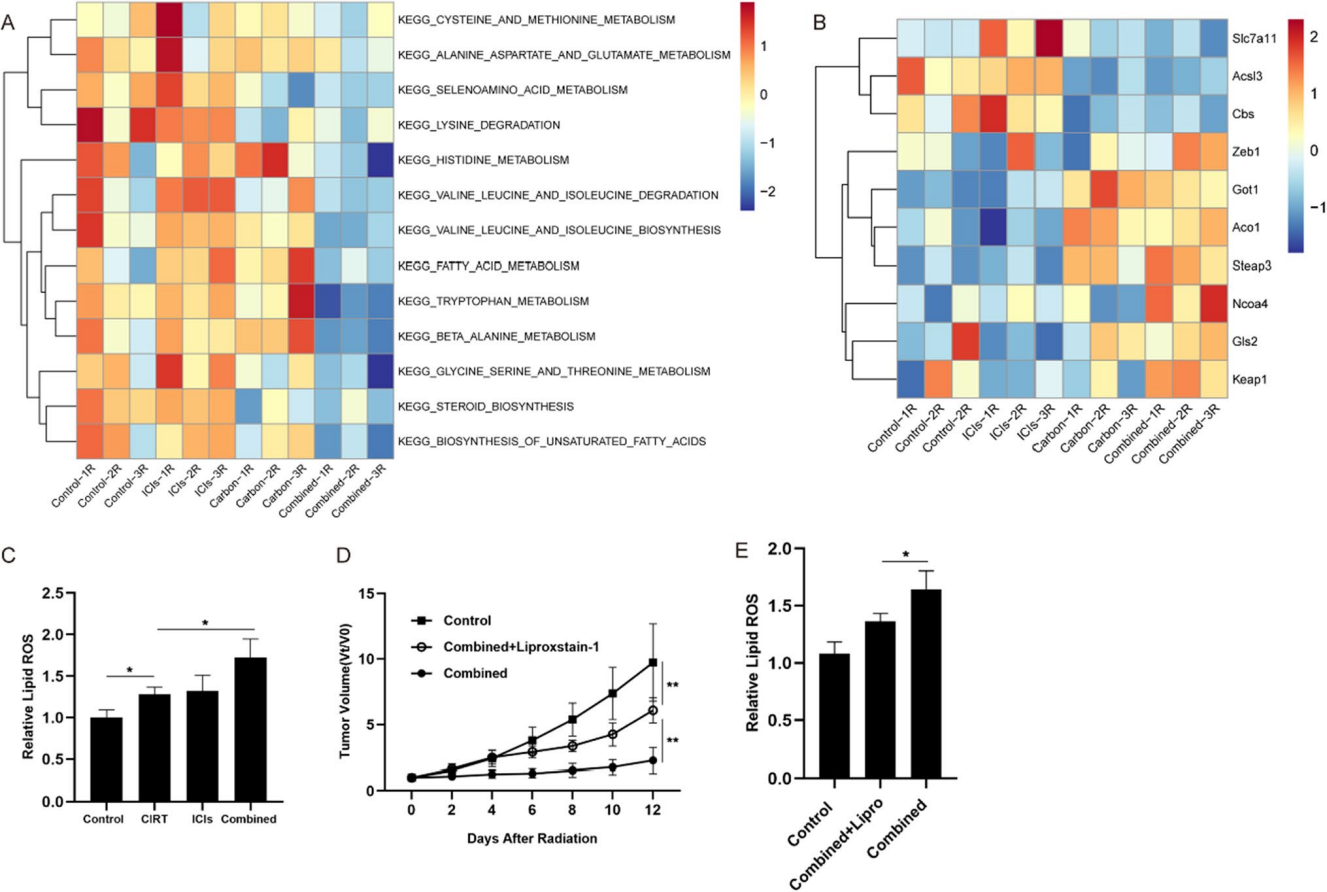
Ferroptosis is involved in the synergistic efficacy of combination therapy. **A** Bioinformatics analyses demonstrated the enrichment of lipid and amino acid metabolism pathways related to ferroptosis in each group. **B** Enrichment of genes related to ferroptosis. **C** The levels of lipid ROS in each group. **D** The progression and fold changes of the irradiated tumor sizes after using liproxstatin-1. **E** The levels of lipid ROS after using liproxstatin-1

We employed bioinformatics analysis based on transcriptome sequencing to determine whether ferroptosis is involved in the synergistic anti-tumor activity of irradiated tumors treated with a CIRT and immunotherapy combination. In the combination therapy, we observed considerable enrichment of lipid and amino acid metabolism pathways, which were significant for ferroptosis regulation (Fig. 6A). Additionally, in the combination group, genes that promote ferroptosis were upregulated, whereas genes that suppress ferroptosis were downregulated (Fig. 6B). Next, flow cytometry was used to assess lipid peroxidation as a functional indicator for ferroptosis. As opposed to other groups, the irradiation tumors in the combination therapy group displayed greater lipid ROS levels (Fig. 6C), suggesting that ferroptosis participated in the combination treatment synergistic effectiveness.

Finally, we investigated the effects of the ferroptosis inhibitor liproxstatin-1 on the combination treatment (CIRT and ICI) in vivo to elucidate the ferroptosis implications in combination therapy. The combination therapy substantially suppressed tumor development, whereas liproxstatin-1 attenuated this impact (Fig. 6D). Moreover, lipid ROS levels decreased in the combination treatment plus liproxstatin-1 group (Fig. 6E). Ferroptosis triggered by these two treatments may regulate the anticancer synergistic impact of CIRT and ICIs.

## Discussion

Due to its physical and biological benefits, CIRT, a cutting-edge radiation therapy, may yield provide superior clinical outcomes for patients with radioresistance. The combination of CIRT and ICI treatments is being investigated to fully exploit the advantages of each treatment and create synergistic anti-tumor effects. [12, 13] Here, we showed that the local and abscopal anticancer effects of carbon ions combined with ICIs were more potent than those of the control, carbon ion alone, and ICIs alone in a mouse model. Bioinformatics results showed that the immunological effect, including the enrichment of immune-related pathways, molecules, and immune cell infiltration, in mouse tumor tissues in the combination treatment group was more pronounced. These results were corroborated by flow cytometry, immunofluorescence, and multi-cytokine assay results and confirmed the degree of immune cell infiltration in tumor tissues, demonstrating that the combination therapy resulted in the greatest immune activation. Finally, we observed that ferroptosis potentially contributes to the anti-tumor synergy elicited by CIRT and ICIs. These results provide a reference point for future investigations into underlying mechanisms, as carbon ion and ferroptosis data have not been previously reported.

Preclinical and clinical data indicate that the combination of innovative radiation and immunotherapy has promise for improving treatment effectiveness and reducing recurrence by increasing the capacity of the immune system to identify and eradicate tumor cells and avoid tumor immune tolerance mechanisms. [21–24] Carbon ions are thought to have synergistic effects considering prior research on photon combination ICIs. In our study, CIRT and ICI therapy produced the slowest tumor development in mice in both irradiated and unirradiated tumors, suggesting that using CIRT with ICIs, similar to photons, may have a synergistic impact on malignancies. The incorporation of carbon ions with ICIs improves the anticancer effect for both local and abscopal cancers and reduces liver and lung metastasis in vivo. [12, 13, 25] The outcomes of these preclinical investigations, including those of our study, support the use of carbon ion combination immunotherapy in clinical settings. Although photon combination immunotherapy has shown improved efficacy in some radiation-resistant cancers, such as recurrent nasopharyngeal carcinoma, there is still space for improvement in the clinical context. [26] We previously discussed the clinical results of 206 individuals receiving CIRT for recurrent nasopharyngeal cancer. The 2-year overall survival (OS) rate was 83.7%, which was a promising result and considerably higher than that of the photon. [27] Carbon ion combination immunotherapy or carbon ion coupled immunotherapy use in medical settings has not been reported. We are currently conducting a phase II clinical trial with ICIs and CIRT for recurrent nasopharyngeal cancer, which is registered at ClinicalTrials.gov (ID: NCT04143984). We anticipate the outcomes of this trial with eagerness and aim to offer therapeutic support for carbon ion combination immunotherapy.

The immune system activates anti-tumor mechanisms via innate and adaptive immunity, which is consistent with the enrichment for immune-related pathways in our on-treatment differential pathway analysis and the enrichment for irradiation tumor-derived DEGs as determined from our transcriptome sequencing data. The degree of immune activation or the function of infiltrating CD8 + T cells in tumors, which are strongly associated with tumor development prevention, is reflected by enriched immune response-related pathways and genes in the combination group. [28, 29] The immune cell infiltration proportion may be used to predict prognosis, survival, and metastasis and gauge the anti-tumor response effectiveness. [30] Using ssGSEA and ESTIMATE immune scoring methodologies, our findings indicated that the combination therapy group had the greatest immune cell infiltration levels. Similarly, CD8 + cell infiltration in the combination treatment group was the most obvious using flow cytometry and immunofluorescence techniques. Certain cells, specifically CD8 + T cells, exhibit immunomodulatory properties in cancer, increasing the anti-tumor immune response, and inhibiting tumor growth and metastasis. [31] Similar investigations revealed that the combination of CIRT and ICI treatment dramatically increases immune cell infiltration compared to single therapy modes, even when compared to the photon RT combined with ICIs. [12, 13, 25] Our application of CD8 monoclonal antibody revealed that the combination therapy group's synergistic anti-tumor effect was reversed, which further demonstrated the significance of CD8 cell infiltration for anti-tumor effects. Additionally, an effective foundation for CIRT combination therapy was also established by earlier research comparing the effects of carbon ions and photons on tumor cell immunogenicity. Carbon ions were found to promote tumor cell immunogenic death more effectively than photons. [32, 33] Thus, we hypothesized that CIRT used in a combination treatment may be superior to photon RT in treating patients with radio-resistant or recurrent cancer. In future studies, we will further investigate the variations in internal mechanisms and anti-tumor synergy induced by carbon ions and photons combined with ICIs at the same physical or biological equivalent dose.

Immune cell cytokine or chemokine production in response to inflammation or tumors is a critical component in managing the immune response. [34] Our findings indicated a more complex tumor microenvironment in the combination therapy group, as seen by increased cytokine and chemokine secretion, particularly those with enhanced anti-tumor effects, such as IL-2 and INF-γ. Among the recognized cytokines that play crucial roles in anti-tumor immunity are IFN-gamma (IFNG) and type I IFN [34], enhancing infiltrating CD8 + T cell-produced IFN-γ can boost tumor immunity, which in turn can trigger tumor retreat, by recruiting inflammatory cells to the tumor microenvironment. Additionally, we showed that CIRT stimulates the type I interferon pathway, which drives an inflammatory pathway that increases dendritic cell (DC) activation and cross-presentation of tumor antigen for T cell priming. [35] Chemokines, like CXCL10 (interferon-induced protein 10) and CXCL11 (interferon-gamma-inducible protein 9), were elevated in the combined therapy group throughout our experiments, which was previously known to be substantially induced by IFN-γ and IFN-β. The CXCL9, -10, -11/CXCR3 axis regulates immune cell migration and activation, including that of CTLs and NK cells, which inhibit tumor development. [36, 37] Therefore, strengthening the combination treatment synergistic impact would be beneficial.

Although radiotherapy in conjunction with ICIs is effective, the mechanism of action is still being investigated to develop a better combination therapy strategy and fully exploit the benefits of both treatments in triggering the immune response. [34, 35] Ferroptosis is an underappreciated factor and a target for developing efficient cancer combination therapy. [15, 18, 38] Therefore, we used bioinformatics analysis to investigate this claim. We observed that the tumor tissues of the combined treatment group displayed significantly greater activation of lipid and amino acid metabolism pathways related to ferroptosis, indicating that ferroptosis may also be involved in CIRT combination treatment. The combination treatment group had the highest ferroptosis level, according to the detection of markers associated with ferroptosis. Further use of ferroptosis inhibitors revealed that the anti-tumor synergistic effect driven by the combination treatment group was hindered in vivo, and the related ferroptosis indicator appeared to be decreased using flow cytometry, preliminary demonstrating that ferroptosis might contribute to the CIRT combination therapy synergistic anticancer effect. Lang et al*.* have found that photon RT and ICIs together suppress SLC7A11 in a manner that promotes tumoral lipid oxidation and ferroptosis, boosting the anti-tumor effect. [15] Radiotherapy can cause ferroptosis by generating ROS and upregulating ACSL4, and certain proteins, such as ACSL4, SLC7A11, and GPX4, are co-regulated with ferroptosis, highlighting the value of ferroptosis in combined radiation and other therapeutic methods. [16] However, the specific molecule that drives ferroptosis in CIRT combined with ICIs has not been reported. The sequencing results here also established variations in the expression of regulatory molecules related to ferroptosis in various groups, such as NOCA4. Future research on the specific molecular mechanism of ferroptosis during CIRT is required.

The abscopal effect, which is the immune-mediated removal of malignancies from a distance from the radiation source, has also been linked to radiotherapy. CIRT alone may also induce the abscopal effect, but the impact is less potent than combination immunotherapy. [12, 19, 25] These findings are in line with ours; the abscopal effect was dramatically diminished when CD8 monoclonal antibodies were used, demonstrating that CD8 + T cells influenced the abscopal effect. However, no precise mechanism of CIRT combination therapy has been identified to cause this phenomenon. By controlling DNA exonuclease *Trex1* gene transcription, varying radiation photon dosages can alter cGAS-STING pathway activation, which in turn affects the type I IFN pathway. This consequence is critical for CD8 T cell priming, which mediates ectopic effects, in combination with ICI treatment. [35] The mechanism of this pathway has not been confirmed in CIRT. Moreover, ferroptosis was identified to be a potential variable in the local anti-tumor synergistic effect produced by CIRT combined with ICIs in our earlier experiments; however, there is no proof that ferroptosis also promotes the abscopal effect. Therefore, further research is warranted to clarify the mechanism underlying the CIRT combination therapy anti-tumor action.

Our findings need to be confirmed using additional tumor models to determine their generalizability and specificity given that this is the first study, to the best of our knowledge, reporting the synergistic anti-tumor activity and involvement of ferroptosis employing carbon ions coupled with immunotherapy. The molecular mechanism through which ferroptosis affects carbon ion radiation needs to be further clarified. Additional research is also needed to investigate the variations in efficacy between immunotherapy combined with carbon ion and photon beams.

In summary, our research demonstrated that CIRT and ICIs improved the anti-tumor immune effect in a mouse tumor model, in both locally irradiated tumors and distant unirradiated tumors. The degree of cell infiltration and cytokine production in tumor tissues were both significantly enhanced by combination therapy, indicating that the therapy may have enhanced the tumor immune microenvironment anti-tumor components. Importantly, ferroptosis may contribute to the onset of anti-tumor synergies in CIRT combination therapy, which provides a new view of its capacity to improve immunotherapy responses. The precise ferroptosis regulation mechanism in CIRT combination therapy requires further investigation.

### Supplementary Information

Below is the link to the electronic supplementary material.

Supplementary file1 (DOCX 23 kb)

## Data Availability

The datasets generated and/or analyzed during the current study are available from the corresponding author on reasonable request.
