# Supplementary material for: Carbon ion radiotherapy combined with immunotherapy: synergistic anti-tumor efficacy and preliminary investigation of ferroptosis
Source: Cancer Immunol Immunother. 2023 Sep 30;72(12):4077–88. doi: 10.1007/s00262-023-03544-x (PMC10700413; doi:10.1007/s00262-023-03544-x)
Supplement: Supplementary file 1 — Supplementary file1 (DOCX 23 kb) [file 262_2023_3544_MOESM1_ESM.docx]

**Supplementary-**

**Materials and Methods**

**Reagents**

Anti-PD-L1 (Clone: 10F.9G2), anti-CTLA-4 = (Clone: 9H10), and anti-mouse CD8 antibodies (Clone: 2.43) for immune checkpoint blockage were purchased from Bio X Cell (West Lebanon NH, USA). Liproxstatin-1, a ferroptosis inhibitor, was purchased from Selleckchem. Antibodies that inhibit BODIPY 581/591 C11 were acquired from Thermo Fisher Scientific (Waltham, MA, USA).

**Cell line experiments**

The mouse B16-OVA cell line used here was purchased from the American Type Culture Collection (ATCC). The murine melanoma cell line B16 gave rise to the OVA-transfected clone known as B16-OVA. The cells were grown in Dulbecco’s Modified Eagle Medium (DMEM) with 10% fetal bovine serum (FBS) at 37 °C in a humidified environment containing 5% CO_2_.

**Mouse tumor model experiments**

The SPHIC's ethical committee authorized all animal experimentation protocols and techniques. We obtained female, six- to eight-week-old C57BL/6 mice from the Shanghai SLAC Laboratory Animal Company. Mice were housed in a specific pathogen-free (SPF) environment. All animal experiments complied with the National Research Council's Guide for the Care and Use of Laboratory Animals. For experiments involving tumor-bearing mice, animals were subcutaneously injected with 5 × 10^5^ B16-OVA cells into the flanks of both hind legs to initiate tumor formation. Before radiotherapy, tumors were permitted to reach a size of 5 × 5 mm and were systematized within 10% variances in tumor volumes. The formula used to determine tumor volume was L × W^2^ × 0.52 (L was the longest diameter and W was orthogonal to L). Following radiotherapy, the volume was assessed every other day until the tumor size reached 10% of the mouse's body weight. The fold change in tumor volume was computed after normalizing the tumor volume at each time point (Vt) to the original volume (V0).

Mice were divided into four groups to examine the potential benefits of using immunotherapy in conjunction with carbon ions: Group I (Control): Control group with appropriate isotype control mAbs (A rat immunoglobulin G (IgG)); Group II (CIRT): CIRT targeting the right tumor; Group III (ICI): Immunotherapy with anti-PD-L1 and anti-CTLA-4 antibodies; Group IV (Combined): CIRT combined with anti-PD-L1 antibody and anti-CTLA-4 antibody. Each group included seven mice. On days 0, 7, and 14 after radiation treatment, mice in the ICI and Combined groups received intraperitoneal injections of 200 µg of anti-PD-L1 and anti-CTLA-4 antibodies. A rat immunoglobulin G (IgG) isotype antibody was applied as a control.

Mice were divided into three groups to investigate CD8+ cell function in combination therapy: Group I (Control); Group II (CD8α): CIRT combined with anti-PD-L1 antibody, anti-CTLA-4 antibody, and anti-CD8 antibody; Group III (Combined). After implanting subcutaneous tumor cells, mice in the anti-CD8 antibody group received 150 µg of anti-CD8 antibody intraperitoneally every four days.

Mice were divided into three groups to examine the ferroptosis impact in combination therapy: Group I (Control); Group II (Combined with ferroptosis inhibitor liproxstatin-1): CIRT combined with anti-PD-L1 antibody, anti-CTLA-4 antibody, and liproxstatin-1; Group III (Combined). After radiation therapy, mice in the ferroptosis inhibitor liproxstatin-1 groups received daily intraperitoneal injections of 30 mg/kg liproxstatin-1.

**Treatment planning and delivery of CIRT**

Single fraction CIRT (4 Gy, physical dosage) was administered to tumors on the right hind legs of the mice. Radiation was delivered according to previously described methods. [19] The energy of the carbon beams ranged from 118.41 to 140.01 MeV/u, and the mean dose averaged linear energy transfer (LET) within the SOBP was 96.94 keV/um.

**RNA sequencing analysis**

RNA differential expression analysis was performed using DESeq2 [20] R package between two different groups. The genes/transcripts with the parameter of false discovery rate (FDR) below 0.05 and absolute fold change ≥ 2 were considered differentially expressed genes (DEGs)/transcripts. Gene Ontology (GO) and Kyoto Encyclopedia of Genes and Genomes (KEGG) studies were carried out to assess functional enrichment to define the biological activities of DEGs. The estimated *t*-test was subjected to FDR correction, with a criterion of FDR ≤ 0.05 employed as the threshold. Tumor collection for RNA-seq was performed 8 days after radiation treatment.

**Bioinformatics analysis of RNA sequencing data**

GSVA R package was used to quantify the signaling pathways in each group, as identified by their enrichment in the MSigDB collection (c2.kegg.v7.1 symbols.gmt; h.all.v7.0.cymbols.gmt). The enrichment score was presented using a heatmap.

The relationship between DEG expression and immune cell infiltration between different treatment groups was analyzed using the single sample gene set enrichment analysis (ssGSEA) method from R package GSVA (version 3.6). These GSEA enrichment scores were then used for each immune cell type that was obtained from each sample and was completed using “GSVA” and “GSEA’’ as the immune cell infiltration measure in each sample. ESTIMATE was used to calculate the immune score, which is the estimate of immune cells in tumor tissue calculated using the “estimate” R package (version 3.6).

**Multi-cytokine assay**

Briefly, tumor tissue was homogenized and cell supernatants from each therapy group were collected for a multi-cytokine test. Cytokine levels were evaluated using a Luminex 200 system (Luminex) using a panel of 31 mouse cytokines (LX-MultiDTM-31) following the manufacturer’s recommendations. The selected cytokines included TNF-alpha, IFN-γ, GM-CSF, IL-10, IL-16, IL-1b, IL-2, IL-4, IL-6, CX3CL, CXCL1, CXCL10, CXCL11, CXCL12, CXCL13, CXCL16, CXCL5, CCL11, CCL12, CCL17, CCL19, CCL2, CCL20, CCL22, CCL24, CCL27, CCL3, CCL4, CCL5, CCL7, and CCL1. Tumors were collected for multi-cytokine tests 8 days after radiation treatment.

**Lipid peroxidation assessment using BODIPY-C11 staining**

A single-cell suspension was created to measure the lipid peroxidation amount in samples from different groups. For B16-OVA tumor-bearing mice, the right subcutaneous tumor tissue was removed, chopped into tiny pieces, mechanically minced against a 100 M cell strainer, and then washed in phosphate-buffered saline (PBS). The cell mixture was collected and density gradient centrifugation was used to pre-enrich tumor and immunological cells. Anti-CD45 and anti-OVA257-264-H2Kb antibodies were used to stain the cell pellet, followed by BODIPY 581/591 C11. After filtration through a 40 M cell strainer, cells were immediately examined using a flow cytometer. The signals from both non-oxidized C11 (PE channel) and oxidized C11 (FITC channel) were observed for BODIPY 581/591 C11 staining. For each sample, the MFI of FITC to the MFI of PE was determined. On other occasions, only the signal from the oxidized C11 was measured, and the MFI of the FITC was computed. The relative lipid ROS results were standardized to control samples.

**Flow cytometric (FCM) analysis of tumor-infiltrating CD45+ and CD8+ T cells**

After the appropriate experimental period, tumor samples from mice were extracted and digested for 30 min at 37 °C with 0.1 mg/mL of DNase I and 1 mg/mL of Collagenase D (Roche). Samples were filtered through a 70-μm cell strainer (MACS SmartStrainers, catalog no. 130-098-462; Miltenyi Biotec, North Rhine-Westphalia, Germany) to obtain the single-cell suspensions, after which they were resuspended and preincubated with Fc-block (catalog no. 553142; BD Biosciences, Franklin Lakes, NJ, USA) to prevent nonspecific Fc-receptor-mediated binding. Cell surface marker staining was conducted using APC-CY7-conjugated anti-mouse CD45 (catalog no. 561037), fluorescein isothiocyanate (FITC)-conjugated anti-mouse CD4 (catalog no. 561828), or BV605-conjugated anti-mouse CD8 antibodies (catalog no. 563152; all antibodies were purchased from BD Biosciences), and BV510-conjugated anti-mouse fixable viability dye (catalog no. 564406) staining was performed on ice in the dark for 40 min. Using a flow cytometer (CytoFLEX S; Beckman Coulter, Pasadena, CA, USA), tumor-infiltrating CD8+ and CD45+ cells were further assessed. Tumors were collected for FCM analysis 8 days after radiation treatment.

**Immunofluorescence assays**

Multiplex immunofluorescence studies using successive staining cycles were performed for tissue immunofluorescence staining. After being deparaffinized, rehydrated, and exposed to antigen retrieval, slides were serially stained with CD4 (1:1000; catalog no. ab183685; Abcam, Cambridge, UK) and CD8 (1:2000; catalog no. ab217344; Abcam)-specific antibodies. Each sample was then treated with anti-rabbit polymeric horseradish peroxidase before being labeled with Opal Fluorophore Reagents. Each antigen was serially tagged with a different fluorophore.

**Statistical analysis**

Two-tailed unpaired Student's *t-*tests or Wilcoxon tests were used to compare the two groups. Two-way ANOVA was used when comparing more than two groups. To determine the *p* values for Kaplan-Meier survival curves, the log-rank test was used. Data are provided as the mean ± SEM. R was used for all bioinformatics statistical calculations (v.3.6.3). The asterisks depicted in the figures indicate significance as follows: **p* < 0.05, ***p* < 0.01, ****p* < 0.001, *****p* < 0.0001 ).
